# Improvement of treatment resistant facial discoid dermatosis with secukinumab

**DOI:** 10.1016/j.jdcr.2025.12.024

**Published:** 2025-12-24

**Authors:** Robin C. Yi, Jennifer J. Su, Matthew R. Powell, Loretta S. Davis

**Affiliations:** a Medical College of Georgia at Augusta University, Augusta, Georgia; b Department of Dermatology, Medical College of Georgia at Augusta University, Augusta, Georgia; c Department of Pathology, Medical College of Georgia at Augusta University, Augusta, Georgia

**Keywords:** facial discoid dermatosis, IL-17 inhibitor, management, secukinumab, treatment options

## Introduction

Facial discoid dermatosis (FDD) is a chronic inflammatory condition characterized by well-demarcated, pink-orange, scaly papules and small plaques on the face and neck.^1^ FDD is often misdiagnosed as seborrheic dermatitis, psoriasis, eczema, or pityriasis rubra pilaris (PRP) due to overlapping clinico-histologic features.^2^ There is currently no standard treatment for FDD, and management remains challenging. Multiple topical and systemic therapies have been reported with variable success including topical corticosteroids, calcineurin inhibitors, vitamin D analogs, retinoids, topical sirolimus, biologics, antibiotics, antifungals, immunosuppressants, and phototherapy.^3^ We report a case of a woman with recalcitrant FDD who improved with secukinumab.

## Case report

**Fig 1:**
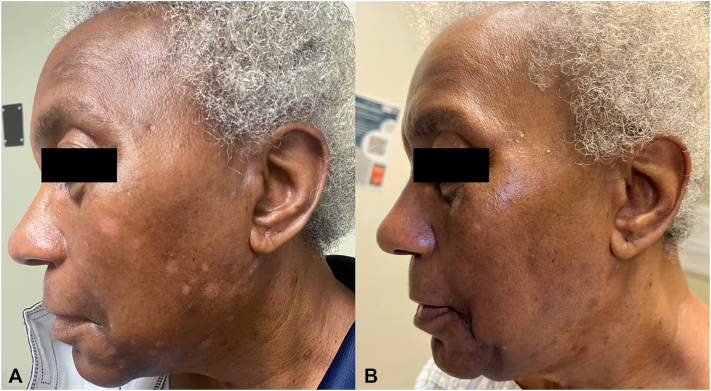
**A,** Multiple well-demarcated, *pink*, scaly papules on the left cheek on methotrexate 15 mg weekly. **B,** Near-complete resolution with residual hyperpigmented macules on the left cheek on secukinumab monotherapy.

**Fig 2:**
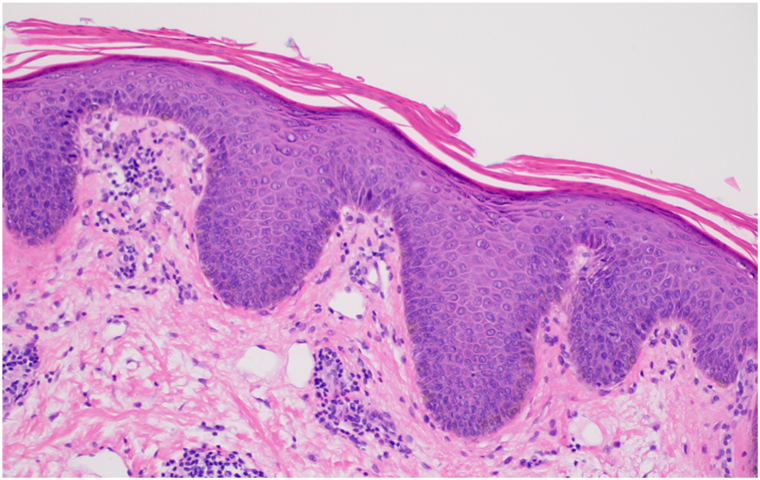
Histopathologic examination reveals broad parakeratosis, mild psoriasiform acanthosis, and mild spongiosis. There is superficial perivascular lymphocytic inflammation without eosinophils (H&E, 200×).

A 72-year-old Black woman presented with a 15-year history of a pruritic facial rash, initially attributed to seborrheic dermatitis. Prior treatments included hydrocortisone 2.5% ointment for over 1 year, triamcinolone 0.1% ointment for 3 months, tacrolimus ointment for over 6 years, ketoconazole cream for over a year, ciclopirox cream for 1 month, and fluconazole 200 mg weekly for 3 months with minimal improvement. Physical examination revealed light pink, scaly papules and small plaques scattered across the cheeks, chin, and neck (Fig 1, *A*). No lesions were observed on the scalp, trunk, or extremities. Biopsy revealed broad parakeratosis overlying psoriasiform epidermal hyperplasia with mild spongiosis and a superficial perivascular lymphocytic infiltrate (Fig 2). Clinicopathologic correlation supported an alternative diagnosis of FDD. Given her recalcitrant condition, systemic therapies were explored. The patient’s insurance required a trial of a systemic immunosuppressant before authorizing a biologic or small molecule inhibitor. Acitretin was not an option due to financial constraints. Methotrexate therapy 15 mg weekly for 6 months and 20 mg weekly for another 6 months provided mild improvement (Fig 1, *A*). Given the limited response to methotrexate, secukinumab 300 mg subcutaneously once weekly for 5 weeks and then every 4 weeks was added to her methotrexate and topical tacrolimus regimen. At 4-month follow-up, the patient reported marked improvement in itch and facial lesions. Physical examination demonstrated rare small, light pink, scaly, thin papules and post-inflammatory hyperpigmented macules (Fig 1, *B*). Methotrexate was tapered off, and improvement was maintained on secukinumab monotherapy for 6 months with no adverse events. Unfortunately, insurance denied further coverage of secukinumab resulting in disease recurrence within 5 weeks following discontinuation of therapy.

## Discussion

FDD is a rarely reported chronic papulosquamous condition first described in 2010.^2^ It typically presents in women in their third decade and is characterized by persistent, round, pink-orange, scaly facial papules and plaques. The pathogenesis of FDD is unknown. Some postulate FDD may be a subtype of PRP (type VII) due to their shared histopathologic features of hyperkeratosis, parakeratosis, psoriasiform acanthosis, follicular plugging, and superficial perivascular lymphocytic infiltrate.^4^^,^^5^ Others propose FDD may be linked to psoriasis or seborrheic dermatitis due to the presence of atrophic sebaceous lobules.^6^ Our case highlights FDD as an under recognized facial dermatosis which can mimic other papulosquamous conditions due to overlapping clinical and histopathologic features. FDD should be considered in patients who do not respond to conventional seborrheic dermatitis or psoriasis therapies.

There is currently no established standard of care for FDD. Of reported treatments, ustekinumab, calcipotriol/betamethasone dipropionate ointment, tacrolimus ointment, and acitretin have the most evidence supporting their use; however, the evidence is limited by the small number of reported cases.^3^ While our patient experienced near resolution of her facial lesions within months of starting secukinumab, 1 reported patient did not experience improvement with either secukinumab or guselkumab but subsequently had partial response to topical sirolimus.^6^ In 2 case reports, ustekinumab therapy resulted in marked improvement of FDD.^1^^,^^7^ These favorable responses suggests that FDD may share pathogenic pathways with other papulosquamous disorders and supports the potential role of biologic therapies as treatment options for FDD.

## Conflicts of interest

None disclosed.
